# Subcortical Brain Volumes Relate to Neurocognition in First-Episode Schizophrenia, Bipolar Disorder, Major Depression Disorder, and Healthy Controls

**DOI:** 10.3389/fpsyt.2021.747386

**Published:** 2022-01-25

**Authors:** Jing Shi, Hua Guo, Sijia Liu, Wei Xue, Fengmei Fan, Hui Li, Hongzhen Fan, Huimei An, Zhiren Wang, Shuping Tan, Fude Yang, Yunlong Tan

**Affiliations:** ^1^Beijing Huilongguan Hospital, Peking University HuiLongGuan Clinical Medical School, Beijing, China; ^2^The Psychiatric Hospital of Zhumadian, Zhumadian, China; ^3^Department of Clinical Pharmacology, Beijing Hospital of the Ministry of Health, Beijing, China

**Keywords:** subcortical, cognitive, MRI, first episode, bipolar disorder, major depression disorder

## Abstract

**Objective:**

To explore differences and similarities in relationships between subcortical structure volumes and neurocognition among the four subject groups, including first-episode schizophrenia (FES), bipolar disorder (BD), major depression disorder (MDD), and healthy controls (HCs).

**Methods:**

We presented findings from subcortical volumes and neurocognitive analyses of 244 subjects (109 patients with FES; 63 patients with BD, 30 patients with MDD, and 42 HCs). Using the FreeSurfer software, volumes of 16 selected subcortical structures were automatically segmented and analyzed for relationships with results from seven neurocognitive tests from the MATRICS (Measurement and Treatment Research to Improve Cognition in Schizophrenia) Cognitive Consensus Battery (MCCB).

**Results:**

Larger left lateral ventricle volumes in FES and BD, reduced bilateral hippocampus and amygdala volumes in FES, and lower bilateral amygdala volumes in BD and MDD were presented compared with HCs, and both FES and BD had a lower bilateral amygdala volume than MDD; there were seven cognitive dimension, five cognitive dimension, and two cognitive dimension impairments in FES, BD, and MDD, respectively; significant relationships were found between subcortical volumes and neurocognition in FES and BD but not in MDD and HCs; besides age and years of education, some subcortical volumes can predict neurocognitive performances variance.

**Conclusion:**

The different degrees of subcortical volume lessening may contribute to the differences in cognitive impairment among the three psychiatric disorders.

## Introductions

Schizophrenia (Sch), bipolar disorder (BD), and major depression disorder (MDD) are severe psychiatric disorders with complex etiology and pathophysiology that are far from being established. These disorders affect millions of people worldwide and are associated with great human and economic costs (stigma, limited activity, decreased life expectancy, and raised healthcare costs). In general, most clinical symptoms of psychiatric disorders, such as delusions, anxiety, irritability, or insomnia, can be effectively treated by current psychopharmacological treatments. Nevertheless, cognitive deficits, which represent core deficits across severe mental disorders, do not improve and can even worsen over time. In Sch, widespread deficits across multiple cognitive domains are well documented (1, 2). Cognitive deficits in BD have been found to be mainly concentrated in attention, verbal learning/memory, and executive function domains (3, 4). Some studies reported that cognitive deficits were particularly related to executive function in MDD (5). Traditional views in neuroscience support the notion that cognitive functioning relies on the neocortical parts of the brain (6), whereas current views suggest that there are associations between cognitive dysfunction and neural distributed networks, including subcortical structures that work in parallel circuits (7).

Subcortical deficits, whether directly or combined with cortical areas, may underline cognitive impairment in normal people and patients with Sch, BD, and MDD (8, 9). Fan et al. demonstrated that the reasoning/problem-solving function was significantly correlated with the volume of the amygdala in first-episode Sch (8). Hartberg et al. (9) reported that bilateral putamen volumes were related to poorer verbal learning, executive functioning, and working memory performance in Sch, and larger left ventricular volumes were related to poorer motor speed and executive functioning in BD.

Meanwhile, previous scientific research has confirmed that subcortical structural abnormalities co-occur with widespread cortical volume reduction even in large-scale studies from the ENIGMA working groups for Sch, BD, and MDD (10–12). The most consistent findings are enlarged lateral ventricles and reduced hippocampal and amygdala volumes in these disorders. However, no studies have so far compared the subcortical structures among these three disorders, despite there being a few studies between Sch and BD or between BD and MDD. Based on previous pieces of evidence of overlapping abnormalities of subcortical structures in the disorders, all similar and different relationships are expected to occur among Sch, BD, and MDD. The purpose of the present study was to explore differences and similarities in relationships between subcortical structure volumes and neurocognition among the four subject groups, including first-episode Sch (FES), BD, MDD, and healthy controls (HCs). We hypothesized that the different degrees of cognitive function impairment in the three disorders were caused by the different degrees of subcortical structure lessening. It meant that the worse the performance of the diagnostic group in cognitive tasks, the smaller the subcortical volume might be.

## Materials and Methods

### Participants

This study included a total of 109 patients with FES (43 men and 66 women), 63 patients with BD (38 men and 24 women), 30 patients with MDD (14 men and 16 women), and 42 HCs (22 men and 20 women). Patients were inpatients and outpatients from the Psychiatric Hospital of Zhumadian (a Zhumadian city-owned psychiatric hospital, Henan Province, China). HCs were also recruited from the local community from Zhumadian with good physical health, and none of them had any positive personal or family history of (or demonstrated) any clinical psychiatric disorders. All subjects were Han Chinese. More details of demographic characteristics for all subjects, as well as clinical and medical information for the patients, are summarized in Table 1.

**Table 1:** Demographic and clinical characteristics of patients with FES, BD, MDD, and HCs.

	**FES** **(*n* = 109)**	**BD** **(*n* = 63)**	**MDD** **(*n* = 30)**	**HCs** **(*n* = 42)**	***χ*** ^**2**^ **/*F***	***P***	***Post-hoc*** **^a^**
**DEMOGRAPHIC INFORMATION**							
Gender(M/F)	43/66	38/24	14/16	22/20	6.61	0.158	
Age (years)	24.4 ± 4.7	27.1 ± 6.4	30.2 ± 5.9	31.9 ± 6.5	16.39	**0.001**	FES>HCs, BD>HCs, FES>MDD
Edu (years)	10.3 ± 2.6	10.1 ± 2.9	9.4 ± 2.3	14.3 ± 2.9	20.88	**0.000**	HCs>FES, BD, MDD
Onset age (years)^b^	23.3 ± 4.7	-	-	-			
Illness duration (years)^c^	0.9 ± 1.2	-	-	-			
Number of manic episodes	-	3.0 ± 1.6	0	-			
Number of depression episodes	-	1.2 ± 0.8	1.8 ± 1.2	-			
**SYMPTOMS**							
YMRS	-	22.5 ± 12.3	3.2 ± 2.9	-			
HAMD	-	9.4 ± 9.6	21.9 ± 6.6	-			
HAMA	-	5.7 ± 7.0	20.2 ± 11.1	-			
PANSS-positive	23.8 ± 7.6	10.4 ± 3.8	-	-			
PANSS-negative	19.4 ± 7.3	7.0 ± 0.0	-	-			
PANSS-general	38.3 ± 9.8	27.8 ± 13.0	-	-			
PANSS total	81.5 ± 21.0	45.2 ± 14.5	-	-			

*FES, first episode schizophrenia; BD, bipolar disorder; MDD, major depression disorder; HCs, healthy controls; M, male; F, female; YMRS, the Young Mania Rating Scale; HAMD, Hamilton Depression Scale; HAMA, Hamilton Anxiety Scale; PANSS, Positive and Negative Syndrome Scale*.

a *Bonferroni post-hoc tests*.

b *Onset age was defined as the time when the patient him/herself or his/her family noticed first symptoms of the disease*.

*Bold font indicates that the p value is less than 0.05*.

The inclusion criteria of FES included the following: (1) Sch diagnosis according to the Diagnostic and Statistical Manual for Mental Disorders—Fourth Edition (DSM-IV) based on the Structured Clinical Interview; (2) male or female patients aged 16 years and older; (3) first outpatient treatment or hospitalization less than 2 weeks; (4) education for at least 6 years; and (5) right-handed confirmation based on the short version of the Edinburgh Handedness Scale. Exclusion criteria were as follows: (1) claustrophobia; (2) a history of head trauma; (3) brain organic disease confirmed by T2 magnetic resonance imaging (MRI); (4) substance dependence or drug abuse now or before; (5) learning disability or mental delay; and (6) other contraindications to MRI.

The inclusion criteria of BD included the following: (1) BD diagnosis and no history of any other Axis I disorder based on the DSM-IV; (2) scores of the Young Mania Rating Scale (YMRS) greater than 13 or scores of the 17-item Hamilton Depression Scale (HAMD-17) greater than 17; (3) male or female patients aged 16 years and older; (4) prescription drugs discontinued at least 2 months before seeking medical advice; (5) education for at least 6 years; and (6) right-handed confirmation based on the short version of the Edinburgh Handedness Scale. Exclusion criteria were the same as those for FES earlier.

Inclusion criteria for patients with MDD were as follows: (1) MDD diagnosis based on the DSM-IV; (2) scores of HAMD-17 greater than 17; and (3) number of depression episodes greater than 2. Other inclusion and exclusion criteria were the same as earlier.

All subjects gave written informed consent and were approved by the Institutional Review Board of the Psychiatric Hospital of Zhumadian. Researchers conducted a detailed questionnaire on each subject, including sociodemographic characteristics, general information, and medical and psychological conditions. More information was collected from available medical records.

### Clinical Procedures

Whether the participants were inpatient or outpatient, they started their treatment without delay. YMRS, HAMD-17, HAS, and the Positive and Negative Syndrome Scale (PANSS) were used to assess the severity of patients' symptoms. Two trained physicians and clinical psychiatrists performed all clinical assessments. The intra-class correlation coefficient (ICC) on these scales between psychiatrists was greater than 0.91. If patients met the inclusion criteria mentioned earlier and the physician considered that the patient was stable enough to participate in an MRI, the patients were asked if they would like to attempt a scan. A complete case report form was filled in after providing written informed consent. An MRI was then scheduled. All patients completed the MRI scan within 2 weeks after starting their medication treatment.

### Image Acquisition

All MRIs were carried out on a GE Signa HDxT 3.0T MRI scanner (GE Medical Systems, LLC, USA). Subjects were placed in a birdcage head coil and individually fitted to a bite bar partially composed of a dental impression compound attached to the coil to reduce head motion. T1-weighted images were acquired by covering the whole brain with a sagittal 3D-MPRAGE (magnetization prepared rapid acquisition gradient echo) sequence: repetition time (TR) = 6.77 ms, echo time (TE) = 2.488 ms, inversion time (TI) = 1,100 ms, field of view (FOV) = 256 × 256 mm^2^, matrix size = 256 × 256, flip angle = 7°, and thickness/gap = 1/0 mm.

### Magnetic Resonance Imaging Data Processing

In this study, subcortical volumes were extracted using Freesurfer software version 5.3.0 (http://surfer.nmr.mgh.harvard.edu/) through a standard procedure, which included motion correction, automated topology corrections, intensity normalization, and automatic segmentation of cortical and subcortical regions, and was documented elsewhere (13, 14). Specifically, the corresponding volumes of eight regions from each hemisphere (17 in total) were chosen, and the total gray volumes were calculated for the investigations, labeled as lateral ventricle, thalamus, caudate, putamen, pallidum, hippocampus, amygdala, nucleus accumbens, and total gray volumes. For quality control, we followed the ENIGMA guideline (http://enigma.ini.usc.edu/): all regions larger than 1.5 or less than 1.5 times the quartile space were identified and visually inspected by overlaying their segmentations on the subjects' anatomical images. A blinded manual check of image quality was conducted to diagnose group identity for: motion artifacts, removal of non-brain tissue and missing brain parts after skull stripping, white matter segmentation, correction of pial surface and any surface that does not follow white/gray matter boundary, and correction of subcortical segmentation caused by ventricular enlargement. Only images whose segmentation was judged to be accurate upon visual inspection were subjected to statistical analyses.

### Cognitive Measures

All subjects completed the MATRICS (Measurement and Treatment Research to Improve Cognition in Schizophrenia) Cognitive Consensus Battery (MCCB) that responds to the need for a reliable, consensus-based set of standards for measuring the change in the cognitive deficits of Sch or other disorders (15, 16) within a week of finishing MRI scanning. The MCCB includes seven cognitive domains: (1) speed of processing: symbol coding, trail making test, part A, category fluency; (2) attention and vigilance: continuous performance test; (3) working memory: spatial span, digit sequencing test; (4) verbal learning: Hopkins Verbal Learning Test; (5) visual learning: brief visuospatial memory test; (6) reasoning/problem solving: mazes; and (7) social cognition: managing emotions. Raw scores were recorded and converted to Chinese-normalized T-scores. Seven-domain T-scores and a composite T-score were calculated.

### Statistical Analyses

All analyses were conducted using SPSS software (version 20.0). Continuous variables first determined the data distribution before statistical analyses. The continuous variables that conformed to the normal distribution were analyzed by variance; the other continuous variables were tested by nonparametric test. The G^*^Power 3.1.9.2 program (http://www.softpedia.com/get/Science-CAD/G-Power.shtml) was used to perform a *post-hoc* power calculation for all difference tests (α = 0.05).

#### Group Differences in Demographic, Clinical, and Subcortical Volume Variables and Neurocognition

Demographic and clinical characteristics were compared across groups using a Student's *t*-test or one-way analysis of variance for continuous variables and chi-square analysis for categorical variables. Group comparisons of neuropsychological variables (T-scores) were made using univariate analysis with age, sex, and years of education as covariates. When subcortical volumes were compared across groups, a univariate analysis was performed, and age, sex, and total intracranial volume (ICV) were controlled. ICV was used as a covariate to account for differences in head size. However, there were two possible reasons for the change of ICV: the first was that it was driven by a smaller subset of regions, and the second was that it appeared to be a global effect that could be dramatically attenuated with adjustment. So, to show the changes of subcortical volumes more comprehensively, a similar analysis unadjusted for ICV was performed. A Bonferroni procedure (adjusted *P* = P × 17 for subcortical volume, adjusted *P* = *P* × 8 for neurocognition) was performed for *post-hoc* comparisons between two groups. Uncorrected *P*-values were reported throughout and followed by an adjusted *P*-value when a test was significant. Adjusted *P* < 0.05 was deemed significant. Effect sizes were calculated using Cohen's d. Correlation analysis between subcortical volumes and neurocognition.

Only the variables that showed statistically significant differences among groups were included in the subsequent analyses. To examine the correlation between subcortical volumes and neurocognition, a correlate analysis was conducted, and Pearson product-moment correlation coefficients were used. First, the correlate analysis was performed for the combined sample, then within each group. The subcortical measures in the left and right hemispheres and T-scores of the seven neurocognitive domains were entered into the analysis with age and ICV as covariates. Adjusted *P* = *P* × 48 (6 significant differences regions × 8 significant differences cognitive parameters) for Bonferroni connection.

#### Group Differences in Correlations Between Subcortical Volumes and Neurocognition

Next, the group differences in correlation between subcortical volumes and neurocognition were compared. The way to do this was by transforming the partial correlation coefficient values from the correlation analysis into z scores. It is also known as Fisher's r to z transformation, and its significance was calculated with an online calculator when two correlation values and different sample sizes were entered (http://www.ocpaz.org/tongji/tongji.html).

#### Effect of Subcortical Volumes on Neurocognition

Given that correlation analysis was the basis and premise of regression analysis, whereas regression analysis was the deepening and continuation of correlation analysis, hierarchical multiple regression analysis using age, sex, and years of education as the first step, subcortical volumes as the second step, was applied to test predictors of subcortical volume change on cognitive performance.

## Results

### Demographic and Clinical Characteristics

The demographic and clinical characteristics are summarized in Table 1. Significant differences were found in age among the groups (*F* = 16.39, *P* = 0.001). *Post-hoc* tests revealed that differences between FES and HCs, between BD and HCs, and between FES and MDD were statistically significant (*P* = 0.014, 0.023, and 0.035, respectively). HCs and MDD were older than FES and BD. There was a significant difference in years of education among groups (*F* = 20.88, *P* = 0.000). *Post hoc* tests showed that HCs had greater years of education than FES, BD, and MDD (*P* = 0.022, 0.026, and 0.001, respectively). There was no significant sex difference between groups (χ^2^ = 6.61, *P* = 0.158).

### Effect Sizes for Group Differences in Subcortical Volumes

**Figure 1:**
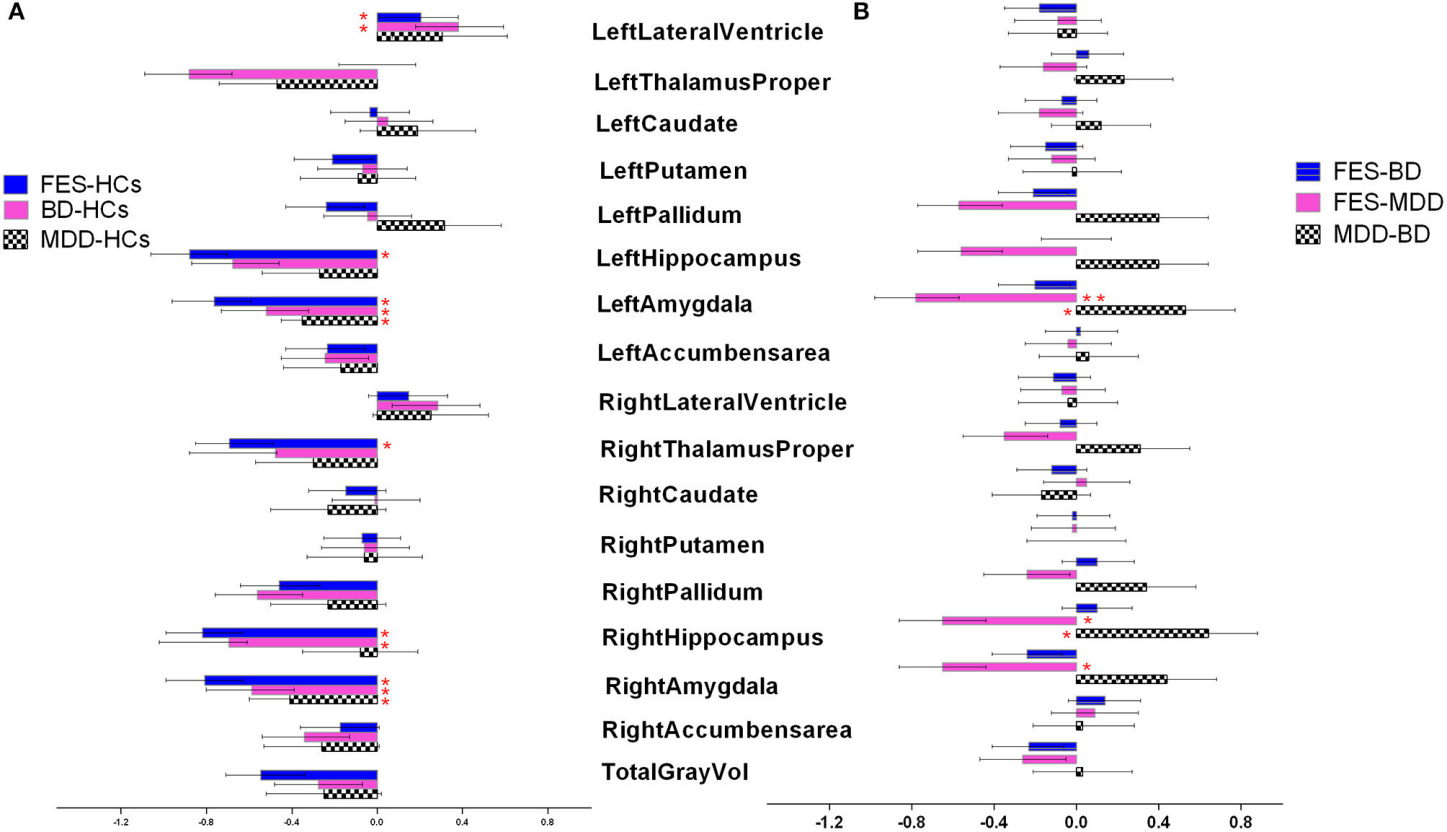
**(A,B)** Cohen's d effect sizes 95% CI and for regional brain volume differences. Effect sizes for all subcortical volumes depicted were corrected for age and intracranial volume (ICV). FES, first episode schizophrenia; BD, bipolar disorder; MDD, major depression disorder; HCs, healthy controls.

Firstly, we assessed case–control differences between each diagnosis group and HCs across 17 brain structures (Figure 1A). A univariate analysis with age, sex, and ICV as covariates showed that left lateral ventricle volumes were significantly larger in FES and BD compared with HCs [Cohen's d (95% confidence interval): *d* = 0.21 (0.02, 0.39), 0.38 (0.18, 0.59), *P* = 4.20 × 10^−4^, 8.7 × 10^−4^, respectively]. After Bonferroni correction, the results were still significant (adjusted *P* = 0.007 and 0.014, respectively). Left hippocampus/amygdala and right hippocampus/amygdala/thalamus volumes were significantly lower in FES compared with HCs [Cohen's d (95% confidence interval): *d* = −0.88 (−1.06, −0.7), −0.76 (−0.96, −0.59), −0.82 (−0.99, −0.63), −0.80 (−0.99, −0.63), −0.69 (−0.85, −0.49), *P* = 5.7 × 10^−5^, 6.3 × 10^−4^, 1.6 × 10^−3^, 3.7 × 10^−4^, 2.8 × 10^−5^, adjusted *P* = 9.1 × 10^−3^, 1.1 × 10^−2^, 2.6 × 10^−2^, 5.9 × 10^−3^, 4.2 × 10^−4^, respectively]. Bilateral amygdala and right hippocampus volumes were significantly lower in BD compared with HCs [Cohen's d (95% confidence interval): *d* = −0.51 (−0.73, −0.32), −0.58 (−0.80, −0.39), −0.69 (−1.02, −0.36), *P* = 4.1 × 10^−4^, 4.3 × 10^−5^, 7.8 × 10^−4^, adjusted *P* = 6.6 × 10^−3^, 6.9 × 10^−4^, 1.2 × 10^−2^, respectively]. When ICV was not adjusted, more significant differences were shown in FES, BD, and MDD compared with HCs. Specific details can be seen in Supplementary Table 1. Secondly, we performed a pairwise comparison with age, sex, and ICV in subcortical volumes among the three diagnosis groups across 17 brain structures (Figure 1B). The results revealed that bilateral amygdala volume and the right hippocampus volume were significantly lower in FES than in MDD [Cohen's d (95% confidence interval): *d* = −0.78 (−0.98, −0.57), −0.65 (−0.86, −0.44), −0.65 (−0.86, −0.44), *P* = 5.7 × 10^−4^, 2.1 × 10^−3^, 1.9 × 10^−3^, adjusted *P* = 9.2 × 10^−3^, 3.4 × 10^−2^, 3.0 × 10^−2^, respectively]. Both the left amygdala volume and right hippocampus volume were significantly lower in BD than in MDD [Cohen's d (95% confidence interval): *d* = −0.53 (−0.77, −0.28), −0.64 (−0.88, −0.40), *P* = 2.1 × 10^−3^, 5.5 × 10^−4^, adjusted *P* = 3.4 × 10^−2^, 8.8 × 10^−3^, respectively]. There was no significant difference in 17 subcortical volumes between FES and BD. When ICV was not adjusted, more significant differences were found between the three diagnostic groups (see Supplementary Table 2).

The statistical power for them is shown in Supplementary Table 3.

### Group Differences in Neurocognitive Tests Among First-Episode Schizophrenia, Bipolar Disorder, Major Depression Disorder, and Healthy Controls

**Figure 2:**
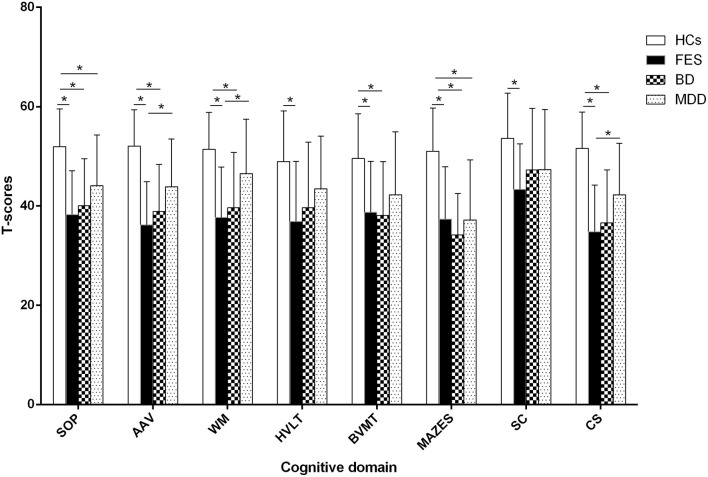
Comparison of cognitive function among groups. * represents Bonfferoni corrected *P* < 0.05/7=0.007. SOP: speed of processing, AAV: attention and vigilance, WM: working memory, HVLT: Hopkins Verbal Learning Test, BVMT: Brief Visuospatial Memory TestM, SC: social cognition, CS: composite T-score, FES: first episode schizophrenia, BD: bipolar disorder, MDD: major depression disorder, HCs: healthy controls.

As shown in Figure 2, a univariate analysis with age, sex, and years of education as covariates revealed that group differences were significant in the T-scores of seven cognitive domains and the composite T-scores (*F* = 53.8, 68.4, 45.5, 24.0, 33.4, 55.5, 21.2, and 77.4, Cohen's *d* = 0.57, 0.61, 0.53, 0.41, 0.47, 0.57, 0.39, and 0.63, all *P* < 10^−4^). Bonferroni *post hoc* tests revealed that the T-scores of seven cognitive domains and the composite T-scores were significantly lower in FES compared with HCs (all *P* < 10^−5^). Patients with BD performed poorer in the speed of processing, attention and vigilance, working memory, visual learning, and reasoning/problem-solving than HCs, and composite T-scores were significantly lower in BD compared with HCs (all *P* < 10^−5^). The T-scores of the speed of processing and reasoning/problem-solving were significantly lower in MDD compared with HCs (both *P* < 10^−4^). The patients with FES had lower T-scores of attention and vigilance, working memory, and composite T-scores than those with MDD (both *P* < 10^−4^). There was no significant difference between FES and BD or between BD and MDD in any test (*P* > 0.05). The statistical power and Cohen's d for group differences are shown in Supplementary Tables 4, 5, respectively.

### Relationships Between Subcortical Volumes and Neurocognition

**Figure 3:**
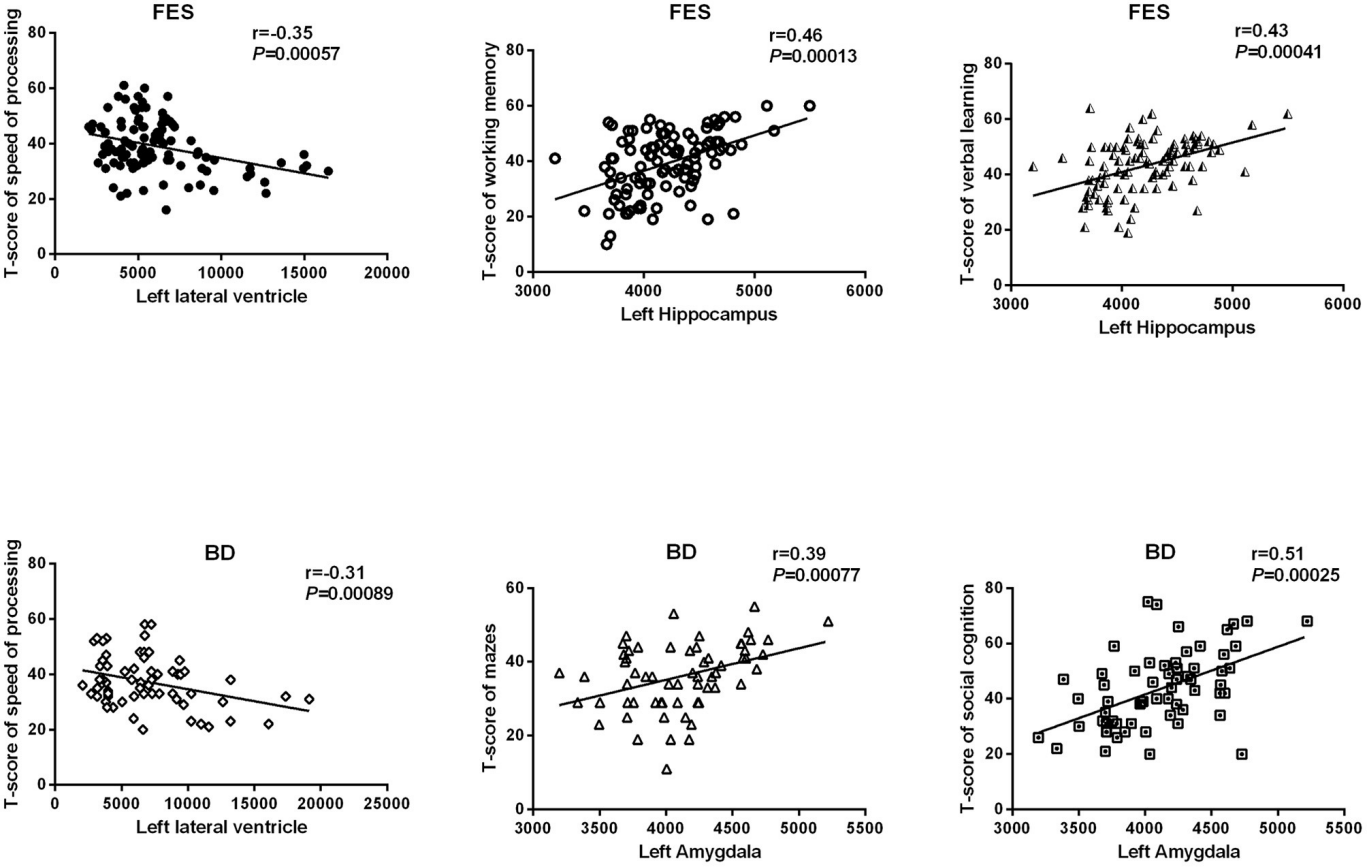
Relationships between subcortical volumes and neurocognition. FES, first episode schizophrenia; BD, bipolar disorder.

The results of the relationships between subcortical volumes and neurocognition are presented in Figure 3. In the FES, significant relationships were found between larger left lateral ventricle volume and lower T-scores of speed of processing (*r* = −0.35, *P* = 5.7 × 10^−4^, adjust *P* = 0.028) and between smaller left hippocampus volume and poorer performances on working memory (*r* = 0.46, *P* = 1.3 × 10^−4^, adjust *P* = 0.006) and verbal learning (*r* = 0.43, *P* = 4.1 × 10^−4^, adjust *P* = 0.020). In BD, larger left lateral ventricle volume was significantly related to poorer speed of processing (*r* = −0.31, *P* = 8.9 × 10^−4^, adjust *P* = 0.044), and smaller left amygdala volume was related to reasoning/problem-solving (*r* = 0.39, *P* = 7.7 × 10^−4^, adjust *P* = 0.038) and social cognition (*r* = 0.51, *P* = 2.5 × 10^−4^, adjust *P* = 0.012). There were no relationships found in the combined sample, MDD, or HCs for any structures and neurocognition (*P* > 0.05/7 regions × 7 neurocognition domains = 0.001).

### Group Differences in Correlations Between Subcortical Volumes and Neurocognition

As shown in Table 2, the correlations between the left hippocampus and working memory in FES were significantly different from BD and HCs (*z* = 2.12, 2.72, *P* = 0.034, 0.0065) and were similar among BD, MDD, and HCs. The correlations between the left hippocampus and verbal learning differentiated the patients with FES from BD and HCs (*z* = 2.13, 2.58, *P* = 0.033, 0.0099), whereas their correlations were similar among BD, MDD, and HCs. The correlations between the left amygdala and social cognition were also significantly different in BD compared with FES (*z* = −2.16, *P* = 0.0091), and the same pattern of relationships was observed between BD and HCs (*z* = 2.39, *P* = 0.017).

**Table 2:** Pairwise contrasts of correlations between subcortical volumes and neurocognitive tests.

	**Group relationships**				**Pairwise contrasts**											
	**FES**	**BD**	**MDD**	**HCs**	**FES-HCs**		**BD-HCs**		**MDD-HCs**		**FES-BD**		**FES-MDD**		**BD-MDD**	
	***r***				**z**	***P***	**z**	***P***	**z**	***P***	**z**	***P***	**z**	***P***	**z**	***P***
Subcortical volume/test																
Left lateral ventricle/SOP	−0.35^a^	−0.31^a^	−0.15	−0.16	−1.09	0.28	−0.77	0.44	0.04	0.96	−0.28	0.78	−0.99	0.32	−0.73	0.46
Left Hippocampus/WM	0.46^a^	0.058	0.11	0.10	2.12	**0.034**	−0.21	0.83	0.04	0.97	2.72	**0.0065**	1.79	0.07	−0.23	0.82
Left Hippocampus/VL	0.43^a^	0.043	0.24	0.06	2.13	**0.033**	−0.08	0.93	0.74	0.46	2.58	**0.0099**	1.00	0.32	−0.87	0.38
Left Amygdala/Mazes	0.23	0.39^a^	−0.039	0.05	0.98	0.32	1.76	0.078	−0.36	0.72	−1.1	0.27	1.27	0.21	1.95	0.05
Left Amygdala/SC	0.14	0.51^a^	0.19	0.07	0.38	0.71	2.39	**0.017**	0.49	0.62	−2.61	**0.0091**	−0.24	0.81	1.60	0.11

*FES, first episode schizophrenia; BD, bipolar disorder; MDD, major depression disorder; HCs, healthy controls; SOP, speed of processing; WM, working memory; VL, verbal learning; SC, social cognition*.

*Bold font indicates that the p value is less than 0.05*.

a *P < 0.05*.

### Effect of Subcortical Volumes on Neurocognition

The hierarchical multiple regression analysis indicated that within the Sch group, age and left lateral ventricle volumes were significant predictors of verbal learning performance (both *P* = 0.000), accounting for 18.3 and 17.7% of verbal learning score variance, respectively. For working memory performance, age, years of education, and right hippocampus volumes were significant predictors (all *P* = 0.000), accounting for 19.4, 11.6, and 16.8% of working memory score variance, respectively.

In the BD group, age, years of education, and left amygdala volume were significant predictors (all *P* = 0.000), accounting for 17.7, 12.2, and 14.9% of working memory score variance, respectively. No significant predictor was found in the MDD group. Table 3 summarizes statistically significant results from the hierarchical multiple regression analysis.

**Table 3:** The hierarchical multiple regression analysis for neurocognition in patients with FES and BD^*^.

**Group**	**Cognitive domains**	**Variable**	**B**	**S.E**.	**95%CI**	**β**	**t**	**Sig**.	**Adjusted R square(%)**	***F***	***p***
FES	Verbal learning	Age	−0.34	0.09	−0.49~-0.19	−0.46	−4.58	0.000	18.3	17.72	0.000
		Left lateral ventricle volumes	0.12	0.01	0.00~0.12	0.43	4.19	0.000	17.7		
	Working memory	Age	−0.55	0.08	−0.87~-0.23	−0.47	−4.65	0.000	19.4	15.48	0.000
		Years of education	0.43	0.11	0.12~0.73	0.29	2.82	0.007	11.6		
		Right Hippocampus volume	0.38	0.00	0.14~0.62	0.49	4.83	0.000	16.8		
BD	Working memory	Age	−0.41	0.06	−0.54~-0.28	−0.39	−5.04	0.000	17.7	16.70	0.000
		Years of education	0.49	0.14	0.19~0.79	0.24	2.74	0.009	12.2		
		Left amygdale volume	0.27	0.02	0.03~0.24	0.45	4.33	0.000	14.9		

*FES, first episode schizophrenia; BD, bipolar disorder*.

* *age, sex and years of education as the first step and subcortical volumes as the second step*.

The statistical power for all analyses is shown in Supplementary Table 3.

## Discussion

The major findings of this study include: (1) larger left lateral ventricle volumes in FES and BD, reduced bilateral hippocampus and amygdala volumes in FES, and lower bilateral amygdala volumes in BD and MDD were presented compared with HCs, and both FES and BD had a lower bilateral amygdala volume than MDD; (2) a comprehensive impairment in seven cognitive dimensions was found in FES, patients with BD had impairment in five cognitive dimensions, and only two cognitive dimensions deficit were shown in MDD, all measured by MCCB; (3) significant relationships were found between subcortical volumes and neurocognition in FES and in BD but not in MDD and HCs; (4) besides age and years of education, some subcortical volumes can predict neurocognitive performances variance. To the best of our knowledge, this is the first study investigating the relationship between subcortical structure and cognitive function in the three disorders and HCs. In addition, the relationships were directly compared across the four groups.

Cognitive impairment is a core feature of neuropsychiatric disorders. More importantly, cognitive impairments persist after clinical remission and lead to social and occupational disability, contributing to the biggest social and economic burden of these neuropsychiatric disorders (17, 18). However, clinical treatment with replicable effects to treat cognitive deficits has not been available so far, one reason of which is that the mechanism of cognitive impairment is poorly understood. The hippocampus may play a key role in cognitive impairment and improvement of cognition due to its critical role in memory and learning. The present study found significantly reduced hippocampus volumes in FES consistent with most similar studies on Sch (19, 20); notably, it was demonstrated that hippocampal volume deficits were more severe in samples with a higher proportion of unmedicated patients in the large sample from the ENIGMA work team (11), indicating that reduced hippocampal volumes were not moderated by the duration of illness or medicine. In our next correlation analysis and hierarchical regression analysis, the relationship between hippocampus volumes and cognitive performance was shown. All these results suggested that the change of hippocampal volumes seemed to be a promising candidate neural marker for cognitive impairment.

It has been suggested that the changes of hippocampal volume in these disorders reflect the decrease of neural plasticity and neurogenesis, which may be downstream effects of abnormally elevated levels of cytokines and cortisol, such as interleukin-1 and tumor necrosis factor-α (21–23). A meta-analysis of blood cytokine network alteration comparison between Sch, BD, and MDD verified that levels of two cytokines (interleukin-6 and tumor necrosis factor-α), one soluble cytokine receptor (sIL-2R), and one cytokine receptor antagonist (IL-1RA) were diversely increased in acutely ill patients with Sch, bipolar mania, and MDD compared with controls (24). Actually, reduced hippocampal volumes were shown not only in FES but also in BD and even MDD in this study (see Figure 1A), despite the biggest effect size in FES. This further confirmed that volumetric hippocampal changes might be associated with aberrant blood cortisol and cytokine levels. In addition, the increase of hippocampal volume may be due to other mechanisms, such as dendritic sprouting or enhancement of brain-derived neurotrophic factor (BDNF) in the hippocampus (21). However, biochemical and immune studies were indeed not involved in the present study. Relevant hypotheses may be verified in further study.

Our other findings included lower bilateral amygdala volumes in all three diagnosis groups compared with HCs, with the biggest effect size in FES and the smallest in MDD. In Sch, a relative certainty was that amygdala volume was reduced compared with HCs. For example, three large-scale meta-analyses showed a statistically significant reduction in the amygdala volume in Sch despite low to moderate effect sizes (d ≈ 0.2) (11, 25–27). However, in BD, there is considerable heterogeneity between studies about amygdala volume. More results showed less pronounced volumetric reductions than in Sch (28, 29), whereas some studies found that the amygdala in BD had more extensive shrinkage than in Sch (30). This discrepancy in findings may stem from differences in medication history, clinical severity and duration, and comorbidities. Differences in neuroimaging data acquisition, amygdala segmentation, and analysis approaches can further affect research results. Few studies have compared subcortical volume between Sch and MDD, but the subcortical comparison between unipolar and bipolar depression has found that the amygdala volume of bipolar depression is smaller than that of unipolar depression (31).

The present study demonstrated that amygdala volume was positively correlated with reasoning/problem-solving and social cognition and predicted 14.9% of working memory performance variance in BD. The amygdala is a fascinating, complex structure that is well known for its involvement in emotion processing, but it has also been documented to be involved in a surprisingly broad array of cognitions, spanning from attention, working memory to long-term memory (32–34). Firstly, the amygdala can regulate the emotional content effect in attention, working memory, and long-term memory. Its mechanism may be realized through the structural and functional connection between the amygdala with the visual cortex, hippocampus, and other brain tissues (35). Among them, the norepinephrine pathway regulates the interaction between the amygdala and hippocampus to complete the memory of emotional content (36). Secondly, the amygdala is involved in difficult working memory tasks, motivational stimulus monitoring tasks, etc. These tasks do not contain any emotional stimuli. Dopamine may play an important role in the interaction between the amygdala and working memory (37). Furthermore, the relationship between the amygdala and cognitive system is bidirectional, which depends on the limbic system and functional connectivity system of the cortical structure (38). Interestingly, hemispheric lateralization seems to appear in FES *vs*. HCs and MDD *vs*. HCs when ICV was not considered, and only effect size between each diagnosis group and HCs was observed, as there were more subcortical structures with significant differences on the left hemisphere (see Supplementary Table 1). Numerous results support the existence of reduced leftward asymmetry in patients with Sch or MDD (39, 40), for example, in the premotor, occipitoparietal, and prefrontal regions for patients with FES (41) or in the cortical–striatal–pallidal–thalamic circuit for patients with MDD (40). Patients with Sch have also been noted to exhibit decreased leftward anatomical asymmetry of the planum temporale or the superior temporal gyrus (42), with some patients even showing rightward asymmetry of the planum temporale (43). Besides brain structural hemispheric lateralization, functional MRI allows the computation of functional cerebral inter-hemisphere differences reflecting the leftward or rightward dominance of activations for a specific task even in the resting state (44). The physiological mechanism of hemispheric lateralization is not well known. Some scholars propose a model in which early life stress and chronic stress not only increase the risk for psychiatric or neurodevelopmental disorders but also change structural and functional hemispheric asymmetries resulting in the aberrant lateralization patterns seen in these disorders. Therefore, pathology-related changes in hemispheric asymmetries are not a factor causing disorders but rather a different phenotype, primarily stress (45).

Several limitations should not be ignored in this study. Firstly, our sample size was not balanced among the groups, especially it was comparatively small in the MDD group, which could lead to false-positive or false-negative results due to the weak statistical power; secondly, this study was not a longitudinal observational study, so it could not provide the course of subcortical volumes with the duration of illness. Thirdly, patients with Sch enrolled in the present study were first-episode patients, whereas neither patients with BD nor MDD were. Actually, if patients with BD were first-episode patients, they had to be undergoing their first manic episode and were in a manic phase. Such a sample in the BD group was limited to the manic phase. However, patients with first-episode unipolar depression clinically showed a relatively high rate of switching to manic in the later stage, but this risk would reduce for patients with recurrent depression. Therefore, to minimize the impact of drugs on patients and consider the comparability with FES, one of our inclusion criteria for BD and MDD was that prescription drugs were discontinued at least 2 months before seeking medical advice.

Based on the results of this study, we found that the effect size was the largest in FES, second in BD, and the smallest in MDD even in the brain regions with no statistical difference between the two groups, suggesting that brain damage may be the most serious in Sch and relatively mild in MDD. We also identified that FES performed the worst in cognitive function, whereas MDD was the best. Combining the relationship between cognitive function and subcortical volumes, these results supported our inference that the difference of subcortical volumes injury may be contributed to the difference of cognitive impairment in three psychiatric disorders.

## Data Availability Statement

The original contributions presented in the study are included in the article/Supplementary Material, further inquiries can be directed to the corresponding authors.

## Ethics Statement

The studies involving human participants were reviewed and approved by the Institutional Review Board of the Psychiatric Hospital of Zhumadian. The patients/participants provided their written informed consent to participate in this study. Written informed consent was obtained from the individual(s) for the publication of any potentially identifiable images or data included in this article.

## Author Contributions

JS and HG appraised potential studies and wrote and revised the draft manuscript. ZW and ST designed the study and revised the draft manuscript. SL, HL, and HA assisted with the presentation of findings and assisted with drafting and revising the manuscript. FF and HF assisted with statistical analysis. FY and YT conceived and designed the study. All authors read and approved the final manuscript.

## Funding

This work was supported by Capital's Funds for Health Improvement and Research (2020-4-2135 and 2018-4-2133), the Beijing Natural Science Foundation (7214238 and 7162087), the Beijing Excellent Talent Traning Program (2016000021469G175), the Beijing Municipal Science and Technology Project (Z181100001518005 and Z171100001017021), the Beijing Municipal Special Foundation for High-Level Health Technology Personnel Construction (PX2016010), Beijing Municipal Science and Technology Commission grant (Z141107002514016), Beijing Municipal Administration of Hospitals Clinical Medicine Development of Special Funding (XMLX201609), and Beijing Hospital Authority Training Plan (PX2018069).

## Conflict of Interest

The authors declare that the research was conducted in the absence of any commercial or financial relationships that could be construed as a potential conflict of interest.

## Publisher's Note

All claims expressed in this article are solely those of the authors and do not necessarily represent those of their affiliated organizations, or those of the publisher, the editors and the reviewers. Any product that may be evaluated in this article, or claim that may be made by its manufacturer, is not guaranteed or endorsed by the publisher.
