# Supplementary material for: Subcortical Brain Volumes Relate to Neurocognition in First-Episode Schizophrenia, Bipolar Disorder, Major Depression Disorder, and Healthy Controls
Source: Front Psychiatry. 2022 Jan 25;12:747386. doi: 10.3389/fpsyt.2021.747386 (PMC8821164; doi:10.3389/fpsyt.2021.747386)
Supplement: Supplementary file 1 [file Data_Sheet_1.DOCX]

Table S1 Cohen’s d and 95% confidence interval between each diagnosis group and health controls

|  | FES-HCs | | | | BD-HCs | | | | MDD-HCs | | |  |
| --- | --- | --- | --- | --- | --- | --- | --- | --- | --- | --- | --- | --- |
|  | Cohen’s d | Upper limit | Lower limit | *P* | Cohen’s d | Upper limit | Lower limit | *P* | Cohen’s d | Upper limit | Lower limit | *P* |
| LeftLateralVentricle | 0.20 | 0.38 | 0.01 | 1.8*10^-3*^ | 0.38 | 0.59 | 0.18 | 2.7*10^-4*^ | 0.30 | 0.61 | 0.07 | 4.1*10^-4*^ |
| LeftThalamusProper | 0.00 | 0.18 | -0.18 | 0.461 | -0.88 | -0.68 | -1.09 | 4.2*10^-5*^ | -0.47 | -0.20 | -0.74 | 8.3*10^-4*^ |
| LeftCaudate | -0.03 | 0.15 | -0.22 | 0.752 | 0.05 | 0.26 | -0.15 | 0.731 | 0.19 | 0.46 | -0.08 | 0.005 |
| LeftPutamen | -0.21 | -0.02 | -0.39 | 1.9*10^-3*^ | -0.07 | 0.14 | -0.28 | 0.672 | -0.09 | 0.18 | -0.36 | 0.801 |
| LeftPallidum | -0.24 | -0.06 | -0.43 | 2.7*10^-3*^ | -0.05 | 0.16 | -0.25 | 0.198 | 0.32 | 0.58 | 0.04 | 5.5*10^-4*^ |
| LeftHippocampus | -0.88 | -0.70 | -1.06 | 4.4*10^-5*^ | -0.68 | -0.46 | -0.87 | 8.3*10^-5*^ | -0.27 | 0.00 | -0.54 | 2.7*10^-3*^ |
| LeftAmygdala | -0.76 | -0.59 | -0.96 | 6.7*10^-5*^ | -0.52 | -0.32 | -0.73 | 3.5*10^-4*^ | 0.08 | 0.35 | -0.19 | 0.71 |
| LeftAccumbensarea | -0.23 | -0.06 | -0.43 | 2.8*10^-3*^ | -0.24 | -0.04 | -0.45 | 1.1*10^-3*^ | -0.17 | 0.10 | -0.44 | 0.003 |
| RightLateralVentricle | 0.15 | 0.33 | -0.04 | 0.004 | 0.28 | 0.48 | 0.07 | 0.008 | 0.25 | 0.52 | -0.02 | 0.009 |
| RightThalamusProper | -0.69 | -0.49 | -0.85 | 7.7*10^-5*^ | -0.68 | -0.47 | -0.88 | 7.6*10^-5*^ | -0.30 | -0.03 | -0.57 | 1.9*10^-3*^ |
| RightCaudate | -0.15 | 0.04 | -0.32 | 0.018 | -0.01 | 0.20 | -0.21 | 0.82 | -0.23 | 0.04 | -0.50 | 0.010 |
| RightPutamen | -0.07 | 0.11 | -0.25 | 0.434 | -0.06 | 0.15 | -0.26 | 0.63 | -0.06 | 0.21 | -0.33 | 0.73 |
| RightPallidum | -0.46 | -0.27 | -0.64 | 5.3*10^-4*^ | -0.56 | -0.35 | -0.76 | 5.6*10^-4*^ | -0.23 | 0.04 | -0.50 | 0.009 |
| RightHippocampus | -0.82 | -0.63 | -0.99 | 5.2*10^-5*^ | -0.82 | -0.61 | -1.02 | 2.4*10^-5*^ | -0.08 | 0.19 | -0.35 | 0.545 |
| RightAmygdala | -0.81 | -0.63 | -0.99 | 3.5*10^-5*^ | -0.59 | -0.39 | -0.80 | 9.3*10^-5*^ | -0.08 | 0.19 | -0.35 | 0.476 |
| RightAccumbensarea | -0.17 | 0.01 | -0.36 | 0.025 | -0.34 | -0.13 | -0.54 | 4.4*10^-4*^ | -0.26 | 0.01 | -0.53 | 0.020 |
| TotalGrayVol | -0.55 | -0.34 | -0.71 | 4.2*10^-4*^ | -0.28 | -0.07 | -0.48 | 1.5*10^-3*^ | -0.25 | 0.02 | -0.52 | 0.271 |

Abbreviations: FES, first episode schizophrenia; BD, bipolar disorder; MDD, major depression disorder; HCs, healthy controls; * *P＜0.05/17*

Table S2 Cohen’s d and 95% confidence interval between diagnosis groups

|  | FES-BD | | | *P* | FES-MDD | | | *P* | MDD-BD | | | *P* |
| --- | --- | --- | --- | --- | --- | --- | --- | --- | --- | --- | --- | --- |
|  | Cohen’s d | Upper limit | Lower limit |  | Cohen’s d | Upper limit | Lower limit |  | Cohen’s d | Upper limit | Lower limit |  |
| LeftLateralVentricle | -0.18 | -0.01 | -0.35 | 0.008 | -0.09 | 0.12 | -0.30 | 0.562 | -0.09 | 0.15 | -0.33 | 0.574 |
| LeftThalamusProper | 0.06 | 0.23 | -0.12 | 0.743 | -0.16 | 0.05 | -0.37 | 0.624 | 0.23 | 0.47 | -0.01 | 0.431 |
| LeftCaudate | -0.07 | 0.10 | -0.25 | 0.662 | -0.18 | 0.03 | -0.38 | 0.726 | 0.12 | 0.36 | -0.12 | 0.583 |
| LeftPutamen | -0.15 | 0.03 | -0.32 | 0.515 | -0.12 | 0.09 | -0.33 | 0.241 | -0.02 | 0.22 | -0.26 | 0.616 |
| LeftPallidum | -0.21 | -0.04 | -0.38 | 2.5*10^-3^* | -0.57 | -0.36 | -0.77 | 7.7*10^-5^* | 0.40 | 0.64 | 0.15 | 6.7*10^-5*^ |
| LeftHippocampus | 0.00 | 0.17 | -0.17 | 0.397 | -0.56 | -0.36 | -0.77 | 5.9*10^-5*^ | 0.40 | 0.64 | 0.16 | 5.4*10^-5*^ |
| LeftAmygdala | -0.20 | -0.03 | -0.38 | 4.5*10^-3^ | -0.78 | -0.57 | -0.98 | 3.1*10^-5*^ | 0.53 | 0.77 | 0.28 | 1.3*10^-5*^ |
| LeftAccumbensarea | 0.02 | 0.20 | -0.15 | 0.415 | -0.04 | 0.17 | -0.25 | 0.533 | 0.06 | 0.30 | -0.18 | 0.372 |
| RightLateralVentricle | -0.11 | 0.07 | -0.28 | 0.334 | -0.07 | 0.14 | -0.27 | 0.341 | -0.04 | 0.20 | -0.28 | 0.261 |
| RightThalamusProper | -0.08 | 0.10 | -0.25 | 0.271 | -0.35 | -0.14 | -0.55 | 8.6*10^-5*^ | 0.31 | 0.55 | 0.07 | 6.6*10^-4*^ |
| RightCaudate | -0.12 | 0.05 | -0.29 | 0.517 | 0.05 | 0.26 | -0.16 | 0.632 | -0.17 | 0.07 | -0.41 | 0.361 |
| RightPutamen | -0.02 | 0.16 | -0.19 | 0.483 | -0.02 | 0.19 | -0.22 | 0.542 | 0.00 | 0.24 | -0.24 | 0.784 |
| RightPallidum | 0.10 | 0.28 | -0.07 | 0.271 | -0.24 | -0.03 | -0.45 | 9.1*10^-4*^ | 0.34 | 0.58 | 0.10 | 5.8*10^-4*^ |
| RightHippocampus | 0.10 | 0.27 | -0.07 | 0.725 | -0.65 | -0.44 | -0.86 | 6.5*10^-5*^ | 0.64 | 0.88 | 0.40 | 1.9*10^-5*^ |
| RightAmygdala | -0.24 | -0.07 | -0.41 | 6.1*10^-4^* | -0.63 | -0.42 | -0.84 | 6.9*10^-5*^ | 0.44 | 0.68 | 0.20 | 4.2*10^-5*^ |
| RightAccumbensarea | 0.14 | 0.31 | -0.04 | 0.384 | 0.09 | 0.30 | -0.12 | 0.421 | 0.03 | 0.28 | -0.21 | 0.192 |
| TotalGrayVol | -0.23 | -0.06 | -0.41 | 5.6*10^-3^ | -0.26 | -0.05 | -0.47 | 5.4*10^-4*^ | 0.03 | 0.27 | -0.21 | 0.536 |

Abbreviations: FES, first episode schizophrenia; BD, bipolar disorder; MDD, major depression disorder; HCs, healthy controls; * *P＜0.05/17*

Table S3 Statistical power for group differences in subcortical volumes

|  | FES-BD | BD-HCs | MDD-HCs | FES-BD | FES-MDD | MDD-BD |
| --- | --- | --- | --- | --- | --- | --- |
|  | *Power* | *Power* | *Power* | *Power* | *Power* | *Power* |
| LeftLateralVentricle | 0.893 | 0.876 | 0.711 | 0.596 | 0.284 | 0.345 |
| LeftThalamusProper | 0.246 | 0.905 | 0.846 | 0.373 | 0.225 | 0.084 |
| LeftCaudate | 0.072 | 0.731 | 0.538 | 0.596 | 0.121 | 0.172 |
| LeftPutamen | 0.804 | 0.672 | 0.370 | 0.119 | 0.241 | 0.427 |
| LeftPallidum | 0.816 | 0.198 | 0.831 | 0.941 | 0.828 | 0.856 |
| LeftHippocampus | 0.897 | 0.949 | 0.796 | 0.119 | 0.831 | 0.804 |
| LeftAmygdala | 0.905 | 0.822 | 0.549 | 0.834 | 0.876 | 0.871 |
| LeftAccumbensarea | 0.794 | 0.807 | 0.409 | 0.916 | 0.507 | 0.562 |
| RightLateralVentricle | 0.782 | 0.391 | 0.770 | 0.625 | 0.562 | 0.126 |
| RightThalamusProper | 0.839 | 0.929 | 0.371 | 0.496 | 0.752 | 0.867 |
| RightCaudate | 0.027 | 0.284 | 0.376 | 0.106 | 0.563 | 0.564 |
| RightPutamen | 0.243 | 0.773 | 0.228 | 0.760 | 0.513 | 0.176 |
| RightPallidum | 0.801 | 0.857 | 0.293 | 0.139 | 0.866 | 0.858 |
| RightHippocampus | 0.876 | 0.828 | 0.521 | 0.642 | 0.845 | 0.906 |
| RightAmygdala | 0.884 | 0.809 | 0.373 | 0.907 | 0.841 | 0.837 |
| RightAccumbensarea | 0.699 | 0.931 | 0.596 | 0.596 | 0.039 | 0.047 |
| TotalGrayVol | 0.930 | 0.832 | 0.354 | 0.850 | 0.838 | 0.281 |

Abbreviations: FES, first episode schizophrenia; BD, bipolar disorder; MDD, major depression disorder; HCs, healthy controls

Table S4 Statistical power for group differences in neurocognitive tests

|  | FES- HCs | BD-HCs | MDD-HCs | FES-BD | FES-MDD | MDD-BD |
| --- | --- | --- | --- | --- | --- | --- |
|  | *Power* | *Power* | *Power* | *Power* | *Power* | *Power* |
| SOP | 0.830 | 0.877 | 0.824 | 0.658 | 0.247 | 0.354 |
| AAV | 0.944 | 0.813 | 0.373 | 0.283 | 0.864 | 0.621 |
| WM | 0.915 | 0.905 | 0.077 | 0.793 | 0.857 | 0.679 |
| HVLT | 0.854 | 0.348 | 0.230 | 0.013 | 0.736 | 0.666 |
| BVMT | 0.877 | 0.890 | 0.558 | 0.642 | 0.428 | 0.421 |
| MAZES | 0.813 | 0.835 | 0.835 | 0.432 | 0.435 | 0.498 |
| SC | 0.905 | 0.285 | 0.251 | 0.787 | 0.473 | 0.216 |
| CS | 0.879 | 0.817 | 0.579 | 0.226 | 0.835 | 0.339 |

Abbreviations: SOP: speed of processing, AAV: attention and vigilance, WM: working memory, HVLT: Hopkins Verbal Learning Test, BVMT: Brief Visuospatial Memory TestM, SC: social cognition, CS: composite T-score, FES: first episode schizophrenia, BD: bipolar disorder, MDD: major depression disorder, HCs: healthy controls.

Table S5 Cohen’s d for group differences in neurocognitive tests

|  | FES- HCs | BD-HCs | MDD-HCs | FES-BD | FES-MDD | MDD-BD |
| --- | --- | --- | --- | --- | --- | --- |
|  | Cohen’s d | Cohen’s d | Cohen’s d | Cohen’s d | Cohen’s d | Cohen’s d |
| SOP | -1.653 | -1.381 | -0.872 | -0.196 | -0.606 | 0.405 |
| AAV | -1.982 | -1.562 | -0.961 | -0.298 | -0.835 | 0.519 |
| WM | -1.545 | 1.244 | -0.522 | -0.182 | -0.838 | 0.624 |
| HVLT | -1.075 | -0.786 | -0.526 | -0.219 | -0.577 | 0.317 |
| BVMT | -1.122 | -1.149 | -0.664 | 0.056 | -0.308 | 0.352 |
| MAZES | -1.410 | -1.969 | -1.310 | 0.334 | 0.013 | 0.292 |
| SC | -1.130 | -0.586 | -0.589 | -0.358 | -0.372 | 0.006 |
| CS | -1.998 | -1.641 | -1.039 | -0.176 | -0.752 | 0.538 |

Abbreviations: SOP: speed of processing, AAV: attention and vigilance, WM: working memory, HVLT: Hopkins Verbal Learning Test, BVMT: Brief Visuospatial Memory TestM, SC: social cognition, CS: composite T-score, FES: first episode schizophrenia, BD: bipolar disorder, MDD: major depression disorder, HCs: healthy controls.
